# Testicular Infarction in a Sickle Cell Hemoglobinopathy Patient: A Case Report

**DOI:** 10.7759/cureus.46177

**Published:** 2023-09-29

**Authors:** Turki Alghamdi, Ahmed A Albassri, Eman F AL-Saleh, Ali Alabandi, Abdulaziz Alhussaini

**Affiliations:** 1 Urology Department, King Fahad Specialist Hospital, Dammam, SAU; 2 Urology Department, Dammam Medical Complex, Dammam, SAU; 3 Histopathology Department, Dammam Regional Laboratory and Blood Bank, Dammam, SAU

**Keywords:** vaso-occlusive events, testicular infarction, testicular torsion, sickle cell complication, acute scrotum

## Abstract

Vaso-occlusive phenomena in sickle cell disease lead to ischemia and possible infarction of the affected organ. We report a case of a 20-year-old Saudi male known to have homozygous sickle cell hemoglobinopathy who was admitted to our institution with abdominal pain. One day post admission, the patient developed left testicular pain. Ultrasound showed decreased echogenicity, and Doppler examination showed absent blood flow in the left testicle. Left radical orchidectomy was done, and histopathological assessment revealed ischemic necrosis with sickled red blood cells (RBCs). A few studies have been reported worldwide suggesting that a vaso-occlusive event is the mainstay mechanism in such cases. This is the first case reported in the Eastern Province of Saudi Arabia.

## Introduction

Sickle cell disease is the most prevalent and frequently diagnosed hemoglobinopathy and carries significant clinical implications. The Eastern Province of Saudi Arabia has the highest prevalence of sickle cell disease among newborns, reaching a population prevalence of 2.6% and a carrier status of 21% [1]. Its complications affect all body parts as a vaso-occlusive crisis with the most affected organs being the brain, lungs, kidneys, and bone [2]. Urogenital complications such as priapism, infertility, hypogonadism, and testicular infarction have been reported [3]. A few cases worldwide reported testicular infarction and segmental testicular infarction in sickle cell disease or trait [4-8]. One case was reported in Saudi Arabia with testicular infarction in sickle cell disease [9]. We are reporting the second case in Saudi Arabia and the first case in the Eastern Province, where it has the most common prevalence of sickle cell disease in Saudi Arabia.

## Case presentation

**Figure 1:**
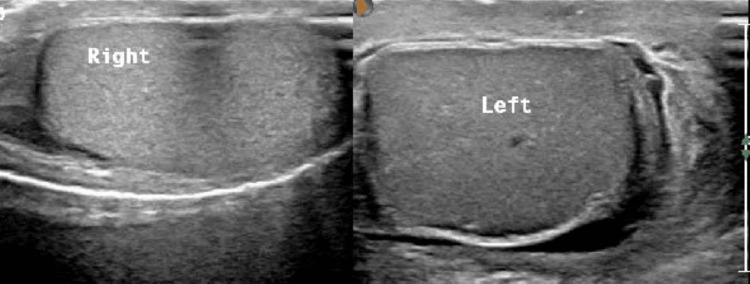
Scrotal ultrasound scan showing hypoechoic left testicle

We report a case of a 20-year-old Saudi male with a known case of sickle cell disease, who is otherwise healthy with no current medications per his medical record. He presented to the emergency room with abdominal pain, mainly in the left lower quadrant. The patient was vitally stable, and his abdomen was tender at the left quadrant. Hemoglobin (Hgb) was 7.4 g/dL, white blood count was 31.1×10^9^/L, reticulocyte count was 5.75%, and hemoglobin electrophoresis showed an Hgb S of 82.9%. Contrast-enhanced abdominal computed tomography (CT) showed splenic mass suggestive of splenic sequestration. The chest X-ray revealed a congested lung suggestive of acute chest syndrome. One day post admission, the patient started to exhibit symptoms of left testicular pain. The patient denied any history of trauma or previous similar episodes of pain. Upon the examination, both testes were swollen more on the left side, with tenderness on fine touch on the left testicle. Scrotal Doppler ultrasound showed that the right testicle is hypervascular, with the left testicle showing no vascularity on color Doppler examination, with mild comparative decreased echogenicity and superadded left hydrocele, raising the suggestion of torsion or an embolism (Figure 1).

**Figure 2:**
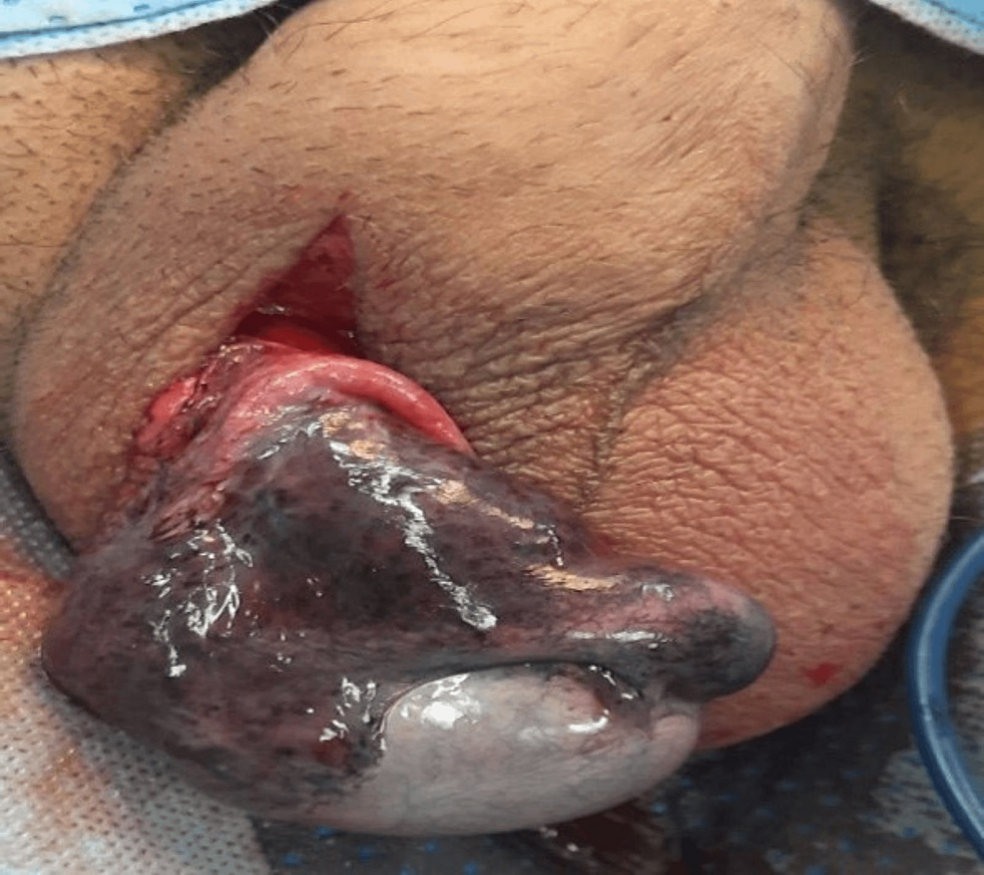
The left testicle showing dark epididymis upon first scrotal exploration

As the clinical and radiological findings suggested, the patient underwent scrotal exploration. No torsion was seen; slight blackish discoloration was noted in the left testis with dense and blackish epididymis. After warming the testis with warm saline, vascularity was seen in the testis and some brightness in the epididymis (Figure 2). The decision was made to keep the testicle and to give the patient the benefit of the doubt. The patient received hydration therapy and an antibiotic course during his hospital stay. After 10 days post scrotal exploration, follow-up scrotal Doppler ultrasonography showed absent blood flow in the left testicle with a mild relative decrease in echogenicity compared to the right side. Radical orchidectomy was offered, but the patient refused despite clearly explaining the outcomes.

**Figure 3:**
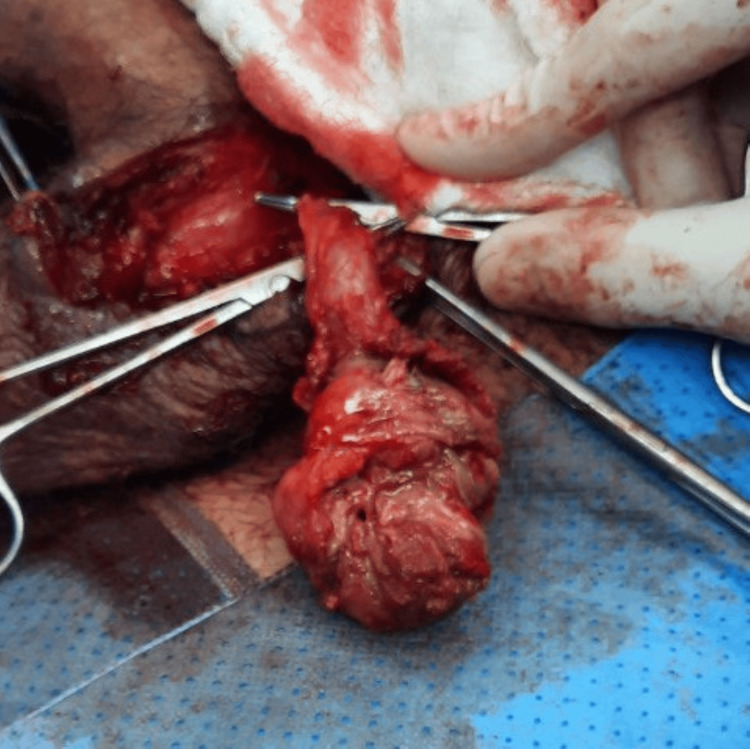
The left testicle showing necrosis upon second scrotal exploration

**Figure 4:**
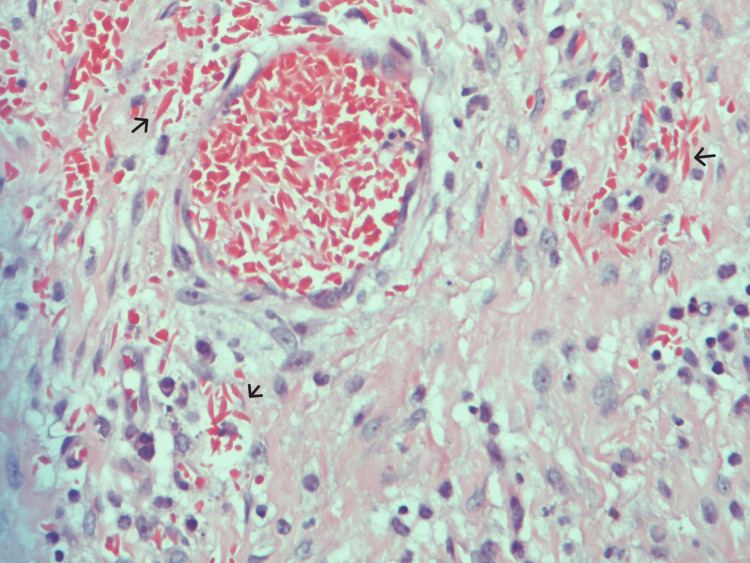
Testicular blood vessel congested with sickled red cells (hematoxylin and eosin {H&E}: ×400)

Twenty days post hospital discharge, the patient presented to the emergency room complaining of discharge from the scrotum and testicular pain. The examination revealed purulent discharge from the wound with the swollen and indurated testicle. The patient agreed to proceed with left radical orchidectomy. Upon second scrotal exploration, a scrotal abscess drained, and the testicle was grossly dark. A radical orchidectomy was performed, and the sample was sent for histopathology (Figure 3). Histopathological evaluation showed ischemic necrosis of the left testicle, and microscopic examination showed sickled red blood cells (RBCs) (Figure 4). The patient was kept for 12 days postoperative to complete the course of ceftriaxone, metronidazole, and vancomycin. Upon follow-up in the clinic, the patient was doing well, and the wound was closed by tertiary intention.

## Discussion

The testes could be a target of vaso-occlusive events in sickle cell patients as other body parts are affected in the sickling process yet less commonly. It might lead to segmental or complete testicular infarction [4-8]. Our patient initially presented with left lower quadrant pain, which then started to be localized to the left testicle. In the other cases reported, patients presented to the emergency room with unilateral scrotal pain [4-9]. Our patient had other vaso-occlusive crisis manifestations, such as acute chest syndrome and splenic sequestration. In contrast, other studies reported patients with only unilateral scrotal pain as their primary complaint [5-9]. Segmental testicular infarction was reported as a patient's first presentation with sickle cell trait undiagnosed before [8].

The histopathological assessment of our patient was consistent with other studies having sickled red blood cells in infarcted testicles [4-7]. The origin of the patients was African American as reported by Li et al. [4] and Holmes and Kane [7], whereas our patient and the patient reported by Alsulmi (2018) [9] were originally from the Eastern Province of Saudi Arabia. The failure of antibiotics and conservative management with such a presentation was reported [7,9]. Another cause of testicular infarction that could present similar to our case is epididymitis [10,11]. Differentiating the underlying cause of unilateral scrotal pain could be challenging as around 70% of the cases reported are idiopathic [12].

## Conclusions

Sickle cell disease could present with testicular infarction. The underlying cause of infarction, whether secondary to testicular torsion or vaso-occlusive crisis, is challenging. We propose that early intervention in the disease process could salvage the testicle; however, research to better define the inflection point at which aggressive treatments versus a more conservative medical treatment or expectant supportive care is sufficient remains urgently needed.
